# Ambulatory ECG Recording in Mice

**DOI:** 10.3791/1739

**Published:** 2010-05-27

**Authors:** Mark D. McCauley, Xander H.T Wehrens

**Affiliations:** Department of Molecular Physiology and Biophysics, Baylor College of Medicine (BCM); The Margaret M. and Albert B. Alkek Department of Medicine, Baylor College of Medicine (BCM)

## Abstract

Telemetric ECG recording in mice is essential to understanding the mechanisms behind arrhythmias, conduction disorders, and sudden cardiac death. Although the surface ECG is utilized for short-term measurements of waveform intervals, it is not practical for long-term studies of heart rate variability or the capture of rare episodes of arrhythmias. Implantable ECG telemeters offer the advantages of simple surgical implantation, long-term recording of electrograms in ambulatory mice, and scalability with simultaneous recordings of multiple animals. Here, we present a step-by-step guide to the implantation of telemeters for ambulatory ECG recording in mice. Careful adherence to aseptic technique is required for favorable survival results with the possibility of implantation and recording from weeks to months. Thus, implantable ECG telemetry is a valuable tool for detection of critical information on cardiac electrophysiology in ambulatory animal models such as the mouse.

**Figure:**
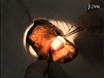


## Protocol

### 1. Preparation of Telemeter for Surgical Implantation

1. Before inserting an ECG telemeter into the mouse, it is important to make sure the ECG telemeter is sterile and in good working order. New telemetry devices are typically provided in a sterile condition by the manufacturer. ECG telemeters can be reused provided the device is cleaned using Tergazyme 1% solution for at least 4 hours. You may rinse the telemeter with sterile water after cleaning with Tergazyme. Additionally, use Wavicide-01 disinfectant overnight to sterilize the ECG telemeter. Be sure to wash disinfectant off with sterile water 48 hours before implantation into mice. Store in a sterile container.
2. Check the telemeter leads for integrity of both the conducting wires and the insulating sheath. Turn on the telemetry transmitter using a magnet waved within 5 cm of the telemeter, and test the signal with an AM radio, frequency 530. The signal should be strong and clear and should vary in intensity and pitch based on manipulation of the wires. Record the model number, the telemeter serial number, and the ECG calibration value. Manufacturers often recommend that the telemeter should be on for 24 hours prior to implantation, see the instruction manual for your specific telemeter for details. In this example, we will use a Data Sciences International (DSI) telemeter for implantation.
3. Telemeter lead preparation may be achieved by cutting the negative (white) lead to approximately 3.5 cm and the positive (red) lead to 2.5 cm. These are the optimal lengths for implantation of the negative lead in the right chest, and positive lead in the left abdomen (see figure 2). Next, remove the insulating sheath to expose 7 mm of wire. Paint the end of the metal lead with sterile superglue, such as Vet-Bond, and then attach lead caps to the metal tips. These caps will avoid skin erosion due to lead placement. Approximately 2-3 mm metal wire should be exposed for electrical sensing of native heart rhythm.

### 2. Surgical Implantation of the Telemeter

1. In order to use sterile technique in murine surgery, you will need sterile gloves, a sterile drape, and 6-0 Prolene sutures. You will also need to 2 pairs of sterilized forceps, 1 pair of blunt-ended scissors, a scalpel, and a needle driver. Surgical instruments may be sterilized in a glass bead sterilizer heated to 250 degrees Celsius. Be sure to sterilize and wash the telemeter as previously described before use.
2. Prepare the mouse for surgery by first anesthetizing the mouse in an induction box using 3% isoflurane in 0.5 L/min 100% O2. Use a heating pad, such as a T-pump, which circulates warm water, to maintain the anesthetized mouse's temperature. Temperature may be monitored by rectal probe, if desired. When the mouse is adequately sedated, completely shave the abdomen and chest of the mouse with electric clippers. Re-anesthetize the mouse with isoflurane before transferring the mouse to a constant-flow tube, supine, with head facing away and tail towards you. Tape down the mouse's paws to the heated surgical table. Wipe the abdomen with an alcohol swab to remove shaved hair and to clean the operative field. Apply three separate coats of betadine with swabs to disinfect the abdomen and chest. Verify correct level of anesthesia by applying pressure on the mouse nail bed. After the mouse has been prepared for surgery, use sterile gloves and apply the sterile drape to the surgical field.
3. Intraperitoneal telemeter implantation offers the advantage of exercise physiology experimentation. For intraperitoneal insertion, begin surgery by using a scalpel to create a vertical midline incision in the skin overlying the abdomen roughly 2.5 cm in length. Carefully separate skin from underlying connective tissues using blunt-ended scissors. Then, create a vertical midline incision in the linea alba overlying the peritoneum, roughly 1.5 cm in length. Additionally, create a small hole in the peritoneum just above (cranial) to the peritoneal incision, which will serve as an outlet for subcutaneous leads. While performing surgery, keep the surgical site moist by occasionally dripping sterile saline onto the operating field.
4. Insert the telemeter into the right peritoneal cavity. Insert forceps into the hole superior to the telemeter and pull both leads up through the lead hole so they are protruding from the peritoneum. Use continuous 6-0 Prolene sutures to close the 1.5 cm peritoneal vertical midline incision.

### 3. Lead Implantation and Abdominal Closure

1. The ECG leads are placed in the lead II configuration. The lead with the white/transparent sheath is negative, and is placed in the left upper abdomen. First, create a 0.5 cm skin incision in the mouse's upper right chest. Next, use the blunt scissors to create a tunnel back to the abdominal incision. Pull the lead through the tunnel and use a 6-0 Prolene suture to anchor the lead to the pectoral muscle. Make sure the suture is on top of the exposed part of the lead, and creates a good contact between lead and underlying muscle. Use a second suture proximal to the aforementioned one, to immobilize the lead to the muscle. Close the skin incision using a 6-0 Prolene suture.
2. The positive lead (red sheath) is placed in the left abdomen below the left diaphragm and below the heart. The lead is anchored to the underlying peritoneal tissue by 6-0 Prolene suture and should have good contact with the peritoneal tissue. An additional incision is not necessary in this step because the site of lead implantation is close to the vertical midline incision, thus may be easily accessed with the existing surgical field. Note that the peritoneal tissue must be lifted to avoid perforation of the underlying intestine.
3. Close the abdominal fascia and skin sequentially with 6-0 Prolene in layers.

### 4. Post-Operative Care

1. Give the mouse 0.1 mL of [1 mg/mL] buprenorphine for analgesia immediately after surgery. Allow the mouse to recover from surgery on a heated pad. If the mouse has had prolonged surgery > 30 min, or if the mouse appears dry, you may inject 0.2-0.3 mL sterile saline into the peritoneum for rehydration. For continued post-operative analgesia it is standard practice to provide mice with buprenorphine twice daily for three days, and then as needed every eight hours.
2. Clean off betadine with an alcohol swab to avoid post-surgical irritation. Remember to record the post-surgical weight for subsequent determination of post-surgical health.
3. 8-24 hours after surgery, give the mouse additional analgesia with buprenorphine when needed, as this will reduce pain and reduce lead placement failure from mouse's clawing at the post-surgical site.

### 5. Representative Results

When performed correctly, the mouse should have closed abdominal incisions with subcutaneous leads under the skin. Usually, the mouse takes 10-30 minutes to recover from anesthesia. It takes about 7-10 days before the mice have completely recovered from surgery, as evidenced by recovery of post-operative weight loss and normalization of mobility.

The most common complication from the surgery is erosion of limb leads through the skin in the first 10 days after the surgery. This common complication may be avoided by firmly adhering subcutaneous leads to subcutaneous tissues or peritoneum. This technique avoids slack leads, which create pressure and friction on the skin when the mouse ambulates. Although less common, infection and sepsis may occur from errors in sterile technique, or from incomplete cleaning of used telemeters. 

ECG output may be recorded by a receiver matrix coupled with data acquisition software, such as Data Sciences International (DSI). Waveform results should include a clearly defined P wave, denoting atrial depolarization, and also a QRS wave which signifies ventricular depolarization, as shown in Figure 3 below. A good quality ECG trace should have a clearly defined P wave preceding a clearly defined QRS wave in a 1:1 ratio. There should be low background signal and R-R intervals should be regular in wild-type mice. A poor quality ECG trace may have indistinct P or QRS waves, or background signal, which may make subsequent computer analysis difficult. A poor ECG trace may be fixed by re-implanting telemeter leads, usually one at a time. To re-implant a telemeter lead, place the mouse under anesthesia, and using surgical technique, make an incision in the skin directly overlying the lead in question. When the lead is accessed, cut the suture anchoring the lead, and move the lead to the desired location before anchoring the lead again. Finally, close the surgical site with 6-0 Prolene. 


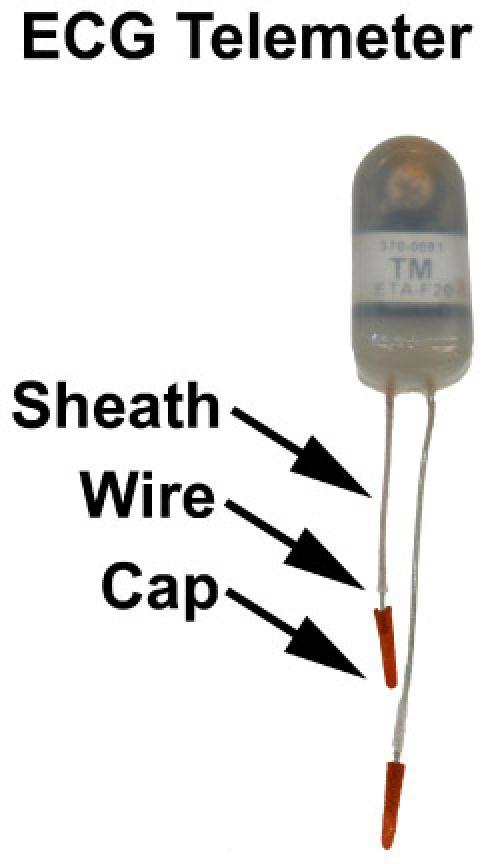
 **Figure 1**: An example of a small animal telemeter with positive and negative leads. In general, the red lead is the positive lead, and the white lead is the negative lead. Each lead should have an insulating sheath, 5-7 mm of exposed wire, and a plastic capped tip, which prevents erosion of the lead through the skin.


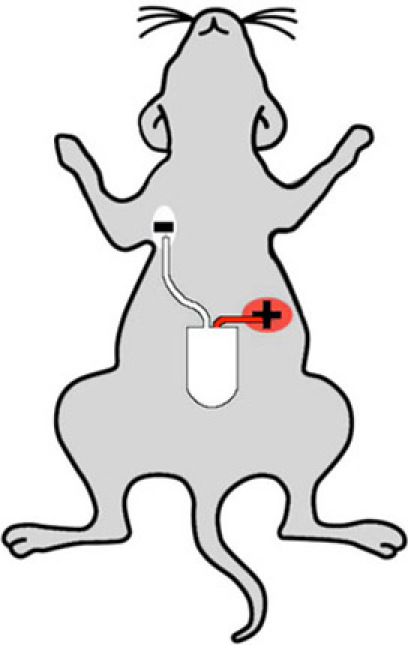
 **Figure 2:** Cartoon showing proper telemeter implantation sites on a mouse. The white (negative) lead is implanted in the mouse's right upper chest, and the red (positive) lead is placed in the left abdomen, such that the telemeter senses the "lead two" configuration.


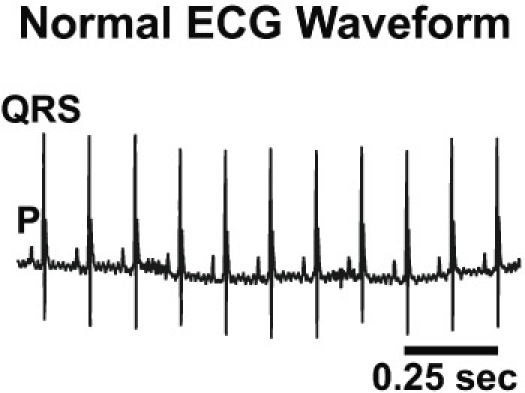
 **Figure 3**: Representative waveform of a mouse in normal sinus rhythm. P wave represents atrial depolarization and QRS complex represents ventricular depolarization. A high quality waveform should have a distinct P wave preceding each QRS complex in a 1:1 ratio.

## Discussion

The critical steps of this procedure are proper cleaning and preparation of the mouse prior to survival surgery. When a telemeter and the mouse operative site are properly cleaned, there is a much better post-surgical outcome. Additionally, close attention to lead placement reduces post-surgical skin erosion. Possible modifications to the technique include alternative configurations to evaluate lateral LV wall function (lead I configuration), or the use of the telemeter not only for ECG waveform analysis, but also for temperature and even blood pressure analysis [beyond the scope of this article]. An alternative location for ECG telemetry recording is subcutaneous lead placement with external wires, i.e. a non-implanted telemeter. This is appropriate for recording of neonatal ECG waveforms, but is not practical for ambulatory ECG telemetry. 

Telemeter implantation is a powerful tool for ambulatory ECG monitoring, and has been used to evaluate the occurrence of ventricular tachycardia, sudden cardiac death, AV nodal block, and atrial fibrillation.^1,2,3^ Additionally, applications of telemetry analysis have yielded valuable data regarding control of heart rate and heart rate variability.^4,5^ Recent advancements in ECG telemeter technology have included sensitive measurements of core body temperature and blood pressure.^6^ Thus, telemeter implantation is a valuable technique for assaying physiological changes in mice and other animal models of disease.
